# Mean Platelet Volume in *Mycobacterium tuberculosis* Infection

**DOI:** 10.1155/2016/7508763

**Published:** 2016-06-22

**Authors:** Min Young Lee, Young Jin Kim, Hee Joo Lee, Sun Young Cho, Tae Sung Park

**Affiliations:** ^1^Department of Laboratory Medicine, Graduate School, Kyung Hee University, Seoul 02447, Republic of Korea; ^2^Department of Laboratory Medicine, School of Medicine, Kyung Hee University, Seoul 02447, Republic of Korea

## Abstract

*Introduction.* Mean platelet volume (MPV) has been thought as a useful index of platelet activation. It is supposed that MPV is also associated with several inflammatory and infectious diseases. Korea still has a high incidence of tuberculosis (TB). The aim of this study was to investigate MPV as an inflammatory marker in TB patients*. Materials and Methods.* MPV were determined in 221 patients with TB and 143 individuals for control group. MPV was estimated by an Advia 2120 (Siemens Healthcare Diagnostics, Tarrytown, NY, USA).* Results.* In the TB patient group, a positive correlation was found between CRP and MPV. Age and MPV had a positive correlation in TB patient group.* Conclusions.* We conclude that there is a significant relation between MPV and inflammatory conditions. MPV can be an inflammatory marker to determine the disease activity in TB patients.

## 1. Introduction

Platelet is well known to be involved in the hemostasis. However nowadays, its different roles are attracting interest, such as actions on inflammation and immunity. Platelets have been widely studied in inflammation-induced atherosclerosis, as well as in thrombosis [1]. Due to the development of the automated complete blood count (CBC) analyzer, platelet indices have been one of the fastest and easiest tests to verify platelet function. Among various platelet indices, the mean platelet volume (MPV) reflects the size of platelets and has been suggested as a useful index of platelet activation [2].

MPV has been also investigated in several infectious diseases such as hepatitis B or mycobacterial infection [3, 4]. Tuberculosis (TB) is one of the most problematic and important diseases threatening public health in Korea. Korea still has a higher prevalence of mycobacterial infection than most other developed countries [5]. Annually, 90 individuals per 100,000 people are newly diagnosed with TB in Korea [6]. Early diagnosis, proper treatment regimen, and determining the activity of TB are important for the regulation of TB. There is no reliable parameter to determining the activity of TB except follow-up of the culture growth of mycobacteria bacilli. A few studies have investigated the relations between TB and MPV [7, 8]. The results were controversial. In this study, we evaluated the MPV in TB patients who were confirmed positive by culture and investigate the meaning of MPV in determining the activity of TB by comparing with C-reactive protein (CRP) as an inflammatory marker.

## 2. Materials and Methods

This study included 221 patients who had positive results on conventional culture tests for mycobacterial species seen at our hospital between January 2011 and April 2012. As the control group, we selected 143 individuals who visited the same hospital for medical check-ups. Extensive chart reviews were done to exclude any individuals with hypertension, diabetes, or smoking from the control group. To ensure that patients had* Mycobacterium tuberculosis* (MTB) infections, the patient group comprised nonoverlapping individuals so that positive results could be identified in a conventional culture study. For solid cultures, 3% Ogawa medium was used. Inoculated medium was incubated for at least 8 weeks at 37°C, in a MGIT 960 incubator (Becton, Dickinson and Company, MD, USA). Culture results were checked weekly. Blood was sampled by venipuncture at antecubital fossa and collected in tubes containing ethylenediaminetetraacetic acid (EDTA). MPV was measured in an Advia 2120 (Siemens Healthcare Diagnostics, Tarrytown, NY, USA) within 2 hours from sampling.

Data were tested for normal distribution using the Kolmogorov-Smirnov test. Statistical comparison was calculated by an unpaired *t*-test. Spearman's coefficient of rank correlation and partial correlation coefficient were used to evaluate the association between MPV, platelet count, CRP, and age. Regression analysis was performed to make regression equation and calculate coefficient of determination. *P* values < 0.05 were considered to indicate statistical significance. The statistical analyses were performed using SPSS version 17.0 (SPSS Inc., Chicago, IL, USA) and Excel 2007 (Microsoft, Redmond, WA).

## 3. Results

The patients' characteristics are summarized in Table 1. The mean age of the TB subjects was 55.86 years, while the mean age of controls was 44.00 years, respectively. Male and female ratio is 1.66 in TB patient group and 1.01 in control group, respectively.

The mean MPV did not differ significantly between the patients (8.03 fL) and controls (7.96 fL). However the platelet count was significantly higher in the TB patients (303 × 10^9^/L) than in the controls (258 × 10^9^/L).

**Figure 1:**
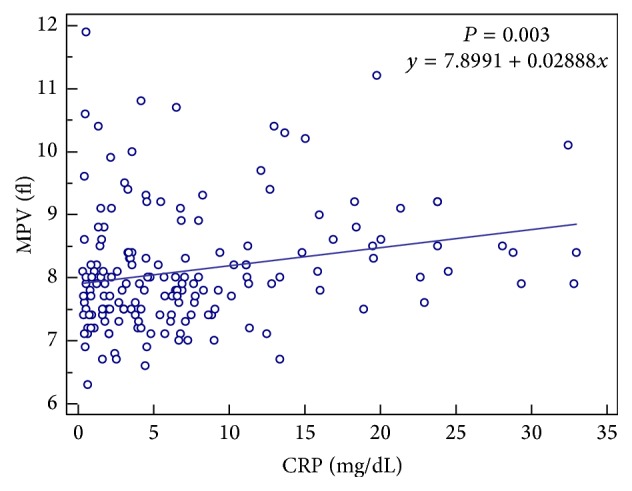
The correlation between mean platelet volume (MPV) and C-reactive protein (CRP) in patients with positive cultures for* Mycobacterium tuberculosis*.

**Figure 2:**
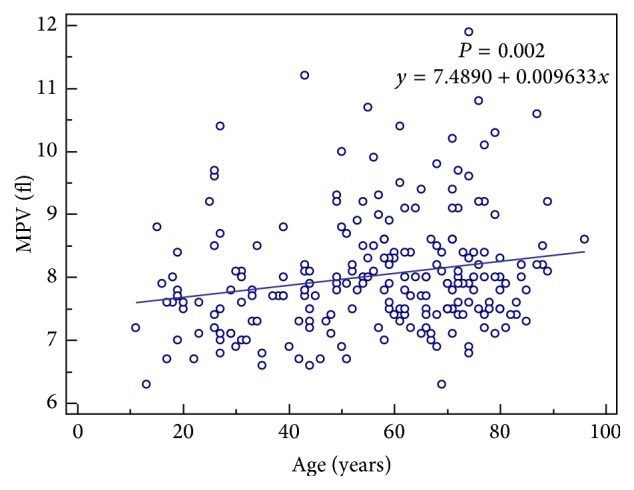
The correlation between mean platelet volume (MPV) and age in patients with positive cultures for* Mycobacterium tuberculosis*.

Among control group, no correlation was found between MPV and the individuals' age, while among TB patient group, positive correlation was found between MPV and patient age (correlation coefficient; rho = 0.235, *P* = 0.002) (Figure 1) and between MPV and CRP (correlation coefficient; rho = 0.206, *P* = 0.002) (Figure 2) by Spearman's coefficient of rank correlation. The partial correlation coefficients of MPV with CRP after adjusting for age are presented in Table 2. However there was no correlation between CRP and platelet count.

## 4. Discussion

Although platelet indices such as MPV have been routinely tested in clinical laboratory using automated hematologic analyzer, their role in the diagnosis and management of diseases has not been fully investigated yet [4, 9]. In platelet study, many investigations have focused on the change of platelet count such as thrombocytosis in inflammatory conditions or its role in hemostasis [10]. Although the function of platelets in hemostasis has been studied thoroughly, more recent evidence has been accumulated in an important role for platelets in the host inflammatory and immune responses [11]. As well as participating in blood coagulation, platelets can act as one of the inflammatory or immune effector cells by releasing inflammatory mediators, activating complement factors, interacting with foreign organisms such as parasites, viruses, and bacteria, and enhancing vascular permeability [1, 2, 10, 11]. Recently, many studies have suggested the importance of MPV as an inflammation marker in some chronic inflammatory disorders, such as rheumatoid arthritis (RA), ulcerative colitis (UC), and psoriasis [2]. However, the results have been controversial and only a few studies have investigated the role of MPV in infection and even fewer have studied the role of MPV specifically in TB infection. To the best of our knowledge, the present study is the largest study investigating MPV as an inflammatory marker in TB patients.

We have found direct relation between MPV and CRP in the TB patient group. As CRP is used commonly as an acute-phase reactant and an inflammatory marker, the result showed that MPV could be used as an inflammatory marker in disease activity assessment in TB. Platelets play a pathophysiological role in making multiple microthromboses around tuberculous cavities to prevent dissemination of the infection [12]. Increasing MPV can be explained by the fact that younger platelets being larger than mature ones are released from bone marrow to the peripheral blood circulation, as platelets are consumed [13]. Recently, it has been reported that various inflammatory conditions increase platelet size and activity [1, 2, 8, 14]. Contrary to our study, Zareifar et al. reported a negative relationship between MPV and serum CRP level. Contradictory results may be related to the characteristics of the studied groups of patients. In the study of Zareifar et al., the subjects were 100 children with all kinds of infectious and inflammatory diseases not defined as acute or chronic. In active phase or attacks of the chronic autoimmune inflammatory disorders, small platelets can circulate dominantly due to the excessively enhanced production of proinflammatory cytokines and acute-phase reactants which decreases the size of platelets [15]. It can also explain the phenomenon that MPV becomes higher after the anti-inflammatory treatment in RA. Gasparyan et al. explained this phenomenon by hypothesizing that high-grade inflammatory diseases, such as active rheumatoid arthritis or attacks of familial Mediterranean fever, result in low levels of MPV because of intensive consumption of large platelet, while low-grade inflammatory diseases or states, such as smoking, diabetes, psoriasis, Behcet's disease, or ankylosing spondylitis, have the opposite effect on MPV because the spleen contains approximately one-third of all the body's platelets which are relatively larger and hemostatically more active than platelets in the systemic circulation [16, 17]. Several studies investigating the relation between MPV and TB have controversial results. Tozkoparan et al. found that the MPV was higher in patients with active TB than in non-TB subjects [14], while Baynes et al. found MPV to be low in patients with active TB [18]. Şahin et al. reported that there was no statistical difference in MPV values between TB patients group and non-TB subjects [8]. However in all those studies, they just compared MPV values of TB patients and non-TB subjects and did not investigate the relation between MPV and CRP.

The effect of age on MPV remains controversial [10]. In this study, in contrast with control group, the increasing tendency of MPV with age was identified in the patient group. We excluded individuals with pathological conditions that may affect MPV values, such as hypertension and smoking from the subjects by means of an extensive chart review. The control group showed no association between MPV and age. This suggests that the platelets of older patients respond more readily to inflammatory or infectious conditions. Aging is known to be associated with increased levels of cytokines and proinflammatory markers. It can result from age-related changes in the immune system and increased secretion of cytokines by adipose tissue [19].

This study had several limitations. Our only inclusion criterion for the patient group was a positive MTB culture; other clinical data, such as medication and duration of disease, were not analyzed. In a future study, these clinical factors should be investigated to rule out their effect on platelet indices.

## 5. Conclusions

In our study we suggest that the changes in MPV are according to CRP and age in TB patients. We found a significant relation between MPV and inflammatory conditions. MPV can be an inflammatory marker measured by the easiest and fastest way to determine the disease activity in TB patients.

## Figures and Tables

**Table 1:** Characteristics of the 221 patients with positive *Mycobacterium tuberculosis* cultures and 143 individuals for control.

Total number of patients	221	143
Mean age (range)	55.86 (11–96) years	44.00 (13–71) years
Correlation between MPV and age^*∗∗*^	rho = −0.067, *P* = 0.426	rho = 0.235, *P* = 0.002
Male : female	138 : 83	72 : 71
Types of specimens cultured		N/A
Sputum	189	
Bronchial fluid	9	
Pleural fluid	8	
Urine	4	
Catheter	3	
Random	1	
Pericardial fluid	2	
Wound	2	
Others^*∗*^	7	

^*∗*^Cerebrospinal fluid, joint fluid, pus, and so forth.

^*∗∗*^By Spearman's coefficient of rank correlation.

N/A, not applicable.

**Table 2:** Partial correlation of MPV with laboratory parameters after adjustment for age.

Variables	MPV (fL)	
	*R*	*P* value
CRP (mg/L)	0.207	0.007
Platelet count (×10^9^/L)	−0.018	0.819

*R*, correlation coefficient.
